# Theories of Change and Mediators of Psychotherapy Effectiveness in Adolescents With Externalising Behaviours: A Systematic Review

**DOI:** 10.3389/fpsyt.2021.730921

**Published:** 2022-01-14

**Authors:** José M. Mestre, Svenja Taubner, Catarina Pinheiro Mota, Margarida Rangel Henriques, Andrea Saliba, Erkki Heinonen, Sara Ramos, Patricia Moreno-Peral, Jana Volkert, Asta Adler, Rasa Barkauskiene, Sonia Conejo-Cerón, Dina Di Giacomo, Yianna Ioannou, Filipa Mucha Vieira, Jan Ivar Røssberg, Célia M. D. Sales, Stefanie J. Schmidt, Tjasa Stepisnik Perdih, Randi Ulberg, Sonja Protić

**Affiliations:** ^1^Instituto para el Desarrollo Social y Sostenible (INDESS), Universidad de Cádiz, Cadiz, Spain; ^2^Institute for Psychosocial Prevention, University of Heidelberg, Heidelberg, Germany; ^3^Department of Education and Psychology, University of Trás-os-Montes and Alto Douro, Vila Real, Portugal; ^4^Center for Psychology at University of Porto, Porto, Portugal; ^5^Faculty of Psychology and Education Science, University of Porto, Porto, Portugal; ^6^Department of Psychiatry, University of Malta and Mental Health Services Malta, Valletta, Malta; ^7^National Institute for Health and Welfare, Helsinki, Finland; ^8^Department of Psychology, University of Oslo, Oslo, Norway; ^9^Instituto de Investigación Biomédica de Málaga, Maálaga, Spain; ^10^Department of Psychology, MSB Medical School Berlin, Berlin, Germany; ^11^Institute of Psychology, Vilnius University, Vilnius, Lithuania; ^12^Department of Life, Health and Environmental Sciences, University of L'Aquila, L'Aquila, Italy; ^13^Department of Social Sciences, University of Nicosia, Nicosia, Cyprus; ^14^Institute of Clinical Medicine, Faculty of Medicine, University of Oslo, Oslo, Norway; ^15^Department of Clinical Psychology and Psychotherapy, University of Bern, Bern, Switzerland; ^16^School of Advanced Social Studies, Nova Gorica, Slovenia; ^17^Department of Psychiatry, Diakonhjemmet Hospital, Oslo, Norway; ^18^Institute of Criminological and Sociological Research, Belgrade, Serbia

**Keywords:** externalising disorders, psychological-treatment effectiveness, externalising behaviours, therapeutic mediation, systematic review, adolescence, distal and proximal factors

## Abstract

**Background:**

Externalising behaviours are becoming a remarkably prevalent problem during adolescence, often precipitating both externalising and internalising disorders in later adulthood. Psychological treatments aim to increase the social functioning of adolescents in order for them to live a more balanced life and prevent these negative trajectories. However, little is known of the intervening variables and mediators involved in these treatments' change mechanisms. We conducted a systematic review, exploring the available evidence on mediators of psychological treatments for externalising behaviours and symptoms amongst adolescents (10 to 19 years old).

**Methods:**

A systematic search was performed on Medline and PsycINFO databases, which identified studies from inception to February 23, 2020. Eligible studies included randomised controlled trials that enrolled adolescents with externalising symptoms and behaviours as, at least, one of the primary outcomes. A group of 20 reviewers from the COST-Action TREATme (CA16102) were divided into 10 pairs. Each pair independently screened studies for inclusion, extracted information from the included studies, and assessed the methodological quality of the included studies and the requirements for mediators, following Kazdin's criteria. Risk of bias of RCTs was assessed by the Mixed Methods Appraisal Tool. Extracted data from the included studies were reported using a narrative synthesis.

**Results:**

Following the Preferred Reporting Items for Systematic Reviews and Meta-Analyses guidelines (PRISMA), after removing duplicates, 3,660 articles were screened. Disagreements were resolved by consensus. In a second stage, 965 full-text articles were assessed for eligibility. A total of 14 studies fulfilled all inclusion criteria. The majority were related to systemic psychological treatment approaches. Two types of mediators were identified as potentially being involved in the mechanisms of change for better social improvements of adolescents: to increase healthier parent–adolescent relationships and parental discipline. However, there were significant and non-significant results amongst the same mediators, which led to discussing the results tentatively.

**Conclusions:**

Family variables were found to be the largest group of investigated mediators, followed by relational, behavioural, and emotional variables. No cognitive or treatment-specific mediators were identified. Both adequate behavioural control of adolescents' peer behaviour and a better positive balance in their relationships with their parents seemed to buffer the effects of externalising behaviours in adolescents. Several methodological limitations concerning mediation testing design, outcome measures, and mediator selection have been identified.

**Ethics and Dissemination:**

Ethical approval was not required. PROSPERO registration number: CRD42021231835.

## Introduction

During childhood and adolescence, externalising, or disruptive behavioural disorders become a significant risk factor for later juvenile delinquency, adult crime, aggressiveness, violence (1), and internalising disorders (2, 3). Both childhood externalising behaviours and juvenile delinquency are currently considered a serious public health concern (4). Consequently, youth violence prevention and intervention are considered one of our society's most pressing issues today (5–7). Identifying essential therapeutic change mechanisms and mediators of outcome in psychological interventions for externalising behaviours problems is of paramount importance as it would help in the treatment and prevention of externalising problems in adolescence and adulthood (8). Previous reviews on psychotherapy with adolescents (10–19 years old) for the treatment of externalising symptomatology have been published to identify potential mediators (6). However, there is still a lack of knowledge of the involved mechanisms of change.

The externalising symptomatology is a category of psychiatric social functioning disturbances (1) that have been categorised as externalising disorders (ED) (9). They can also be present in other specific disorders such as conduct disorder (CD), oppositional defiant disorder (ODD), attention deficit hyperactivity disorder (ADHD) (10), and substance abuse (11). Essentially, almost all ED are characterised by under-controlled (12), impulsive (13), or/and aggressive (14), or rule-breaking behaviours, which have been labelled as externalising behaviours (13) or externalising symptomatology (15).

Traditionally, Achenbach and colleagues introduced the construct of externalising vs. internalising disorders (ID). ED are oriented toward behavioural maladjustment, which affects social functioning. That is, externalising behaviours involve acting negatively on one's external environment. Conversely, ID are oriented toward explaining maladjustment in the child and adolescent's personal functioning (16, 17). However, ED and ID have a substantial overlap, as they share common aetiologies (18). For example, long-term externalising behaviours can lead to internalising symptoms later in life (19, 20) and *vice versa* (21) and are highly correlated (22). Despite the comorbidity of ED and ID, distinctive pathways have been described for externalising disorders from ODD *via* CD to anti-social-personality disorder in adulthood (22).

Some authors have pointed out a lack of consensus on including ADHD and drug abuse in the same category as oppositional defiant disorder and conduct disorder (23). ODD and CD are related more to disruptive behaviours, while ADHD is related to inattention, and alcohol abuse to dopaminergic problems of addiction (17). As stated in the DSM-5 (24), ODD co-occurs with ADHD in approximately half of the cases in the general population in children with a combined presentation (inattention and hyperactivity-impulsivity) and roughly a quarter of children and adolescents with a predominantly inattentive presentation. CD occurs in about one-quarter of children and adolescents with a combined display [e.g., ADHD, see (5)], depending on the age and setting (25). The impulsivity trait that typically manifests in children with ADHD confers considerable risk for the manifestation and development of externalising behaviours (26). Therefore, ADHD is highly comorbid with these disorders and predicts externalising behaviours but is not a disorder caused by the same distal (e.g., parenting style) or proximal (e.g., personality traits) causes (5, 27). Besides, from a neurological point of view, ADHD is considered a neurodevelopmental disorder linked with frontal lobe maturity (28) and substance abuse underlies neurobiochemical mechanisms (29). For the purpose of this review, we have decided to focus only on externalising behaviours and we therefore excluded those articles with a primary diagnosis of ADHD when externalising symptoms were not part of the primary outcomes.

Psychological treatments need to be tailored to the specific needs of individuals with ED, as most psychological interventions have been developed for ID and do not sufficiently address ED (6). Moreover, treatment response and motivation to change may be altered in individuals with ED in comparison to ID (22). Thus, there is a call for a deeper understanding of the mechanisms of change in ED treatment that could inform the development of more effective psychological therapies.

Meta-analyses have concluded that psychological treatments have a more substantial positive effect on externalising symptomatology than punishment-oriented or pedagogical interventions (30). Several psychological therapies have been designed for adolescents' externalising behaviours. The most effective programs include the family and/or peer systems such as multi-systemic (family) therapy, parent training, and multidimensional foster care (24, 26). Third-wave treatments have recently been modified for ED, such as dialectic behavioural treatment (31) and mentalisation-based treatment (32). However, different therapies are based on diverse change theories for addressing externalising symptoms and behaviours among adolescents. Hence, we are interested in reviewing therapies that may have different significant mediators related to externalising outcomes.

The following are the most commonly used psychological therapies for externalising symptoms and behaviours:

- Parent Management Training (PMT): Parent management training is based on behavioural and social learning principles (20, 21). PMT addresses problematic parent–child interactions, especially coercive family processes (33). According to Forehand et al. PMT's mechanisms of change focus on parents learning to replace these problematic interactions with more adaptive ones. Hence, parents are trained to improve positive contingencies to increase the child's desirable behaviours. Parents also receive instructions on applying effective parenting to reduce the child's undesirable behaviours (27). PMT has been recognised for its effectiveness in reducing externalising behaviours (34) and symptomology (35, 36).
- Multisystemic Therapy (MST): Von Sydow et al. described multisystemic therapy (MST) as follows: (1) perceives behavioural and mental symptoms within the context of the social systems in which people live; (2) focuses on interpersonal relationships and interactions, social constructions of realities, and causality between symptoms and interactions; (3) involves family members and significant others (e.g., teachers, friends, other professionals) directly or indirectly; and (4) uses clients' views of problems, resources, and preferred solutions (37). Von Sydow et al. (37) systematically reviewed 47 trials on the efficacy of different psychological treatments for ED in childhood and adolescence. They concluded that, primarily, systemic (family) therapy is effective for externalising behaviours and juvenile delinquency. Furthermore, they noted that systemic therapy effectively affects multiple functioning domains (primary and secondary mental health symptoms, family outcomes, problems with the judicial system, and school performance) (38). The family systems emphasise how family therapy perspectives locate the problem in the workings of the system rather than at the level of the individual (27, 33).
- Cognitive Behavioural Therapy: Cognitive behavioural therapy (CBT) interventions for children and adolescents with clinical symptoms of ED can be either youth-focused (mainly) or parent-focused. CBT aims to improve children and adolescents' coping skills in the face of life's challenges (39). CBT interventions have proven to help children learn to recognise and solve problems (40). When CBT was focused on parents, the primary goal was to improve parents' pedagogical skills, such as rule setting, consistent discipline, or positive reinforcement (41). Conversely, for adolescents and young adults, CBT is recommended when difficulties with socio-cognitive skills are identified in young people with antisocial behaviour problems (42–44). Young people with ED tend to have hostile attribution biases, misinterpret social cues, and have higher expectations of positive outcomes through aggression (34).
- Multidimensional Foster Care Treatment (MTFC): MTFC (45) treats ED children and adolescents with their families with low level of support due to high levels of abuse and neglect, severe mental health and behavioural problems, and juvenile delinquency issues (46). MTFC focuses on young people with severe and chronic delinquency problems. MTFC is a behavioural treatment alternative to residential placement for youth who have problems with chronic antisocial behaviour, emotional disturbance, and delinquency (46). This ED intervention establishes fair and consistent boundaries, supervision, predictable consequences for non-compliance, a supportive relationship with at least one adult mentor, and limited exposure and access to delinquent peers (47). Thus, the MTFC's primary goals are to decrease delinquent behaviour and increase participation in developmentally appropriate prosocial activities for these young people (25). Different MTFC programmes have been developed and validated for older children and adolescents involved in the juvenile justice system (46). MTFC is considered an evidence-based intervention. Several randomised clinical trial studies have shown satisfactory outcomes in treating ED symptoms (48).

All these therapies have highlighted the role of parenting styles as a mediating factor between treatments and outcomes compared to control groups to reduce externalising symptoms in adolescents (27, 35, 36). In summary, the different therapies address parenting styles to treat ED in adolescents. On the one hand, parental warmth, behavioural control, autonomy granting, and democratic parenting styles predict lower ED rates. On the other hand, strict control, authoritarian, permissive, and neglectful parenting styles are associated with more severe externalising problems, with stronger associations observed for stringent control and psychological control (49). However, there is a need to clarify how the theories of change and other mediators in therapies are involved in reducing ED in adolescents. Further research is needed to identify whether more pertinent mediators between treatments and outcomes with a comparator group exist. To our knowledge, there are no systematic reviews on general mediators (beyond parenting styles), both treatment-specific (e.g., psychotherapeutic intervention) and non-treatment specific (e.g., adolescents' capacities for affect regulation), which are involved in psychological treatments to reduce ED symptoms among adolescents. Even though the most common therapies originate from different therapeutic approaches, they overlap in addressing family factors, but they tended to neglect other potential mediators.

### Rationale

This review aims to provide a systematic and comprehensive narrative synthesis of existing studies on mediators (treatment and non-treatment-specific) in psychotherapy with adolescents (10–19 years old) diagnosed with externalising behaviours (impulsivity, disruptive behaviours, aggression or violence, sexual offending, and delinquent behaviours). We aim to identify possible commonalities rather than differences in mediators and theories of change in the diverse interventions where efficacy was evaluated with at least one control group.

## Method

### Study Design

This article is based on work from COST Action 16102 European Network on Individualised Psychotherapy Treatment of Young People with Mental Disorders, supported by COST (European Cooperation in Science and Technology). TREATme was established in 2017 and composed of researchers from 30 countries. TREATme's main objective is to identify scientifically sound empirical research on therapeutic efficacy in young people. Several working groups have been set up to identify mechanisms of change, mediators, and moderators on the therapeutic efficacy, among other activities. This study is a result of work on mediators and theories of change in psychotherapy with adolescents with ED.

This systematic review of the literature follows Preferred Reporting Items for Systematic Reviews and Meta-analyses (PRISMA) guidelines (50). The PICO model defined the research question (patient/population, intervention, comparison, and outcomes); “In adolescents treated for externalising disorders (P), what were the mediators of psychological interventions (I) compared to the effect on the outcomes in other interventions or control groups (C) on ED (O) (51).

### Eligibility Criteria

All full-text versions of potentially relevant studies were searched from inception on February 23, 2020, and were examined in detail for eligibility at the review team meetings. We considered published studies and grey literature from all geographical locations if written in English. Studies were included or excluded in this systematic review based on the following criteria:

### Inclusion Criteria

The inclusion criteria used for the studies were as follows: (1) the study targeted an adolescent sample with a mean age between 10 and 19 years and standard deviation of 3 or lower; (2) the adolescents presented with EDs; (3) the study included a psychosocial intervention and/or psychotherapeutic intervention or treatment for adolescent's ED; (4) the study included a mediating analysis of the change of the intervention; and (5) the study was a randomised controlled trial or controlled study.

### Exclusion Criteria

Articles were excluded if (1) the age of the participants was not given or if the age was below or above the target population; (2) the diagnosis of externalising symptoms was not one of the primary diagnosis; (3) a psychosocial, psychological/psychotherapeutic intervention was not included; (4) mediators were not investigated; or (5) the outcome of the study was not clearly defined or insufficient details were provided to determine whether the outcome was directly related to the intervention.

### Search Strategies

The current study was conducted following the Cochrane Collaboration guidelines for systematic reviews and meta-analyses. The literature search included MEDLINE and PsycINFO databases. All searches were carried out on the same day (February 23, 2020) to control for daily updates. The entire search string is available on https://www.crd.york.ac.uk/PROSPEROFILES/248959_STRATEGY_20210414.pdf.

### Study Selection Processes

**Figure 1:**
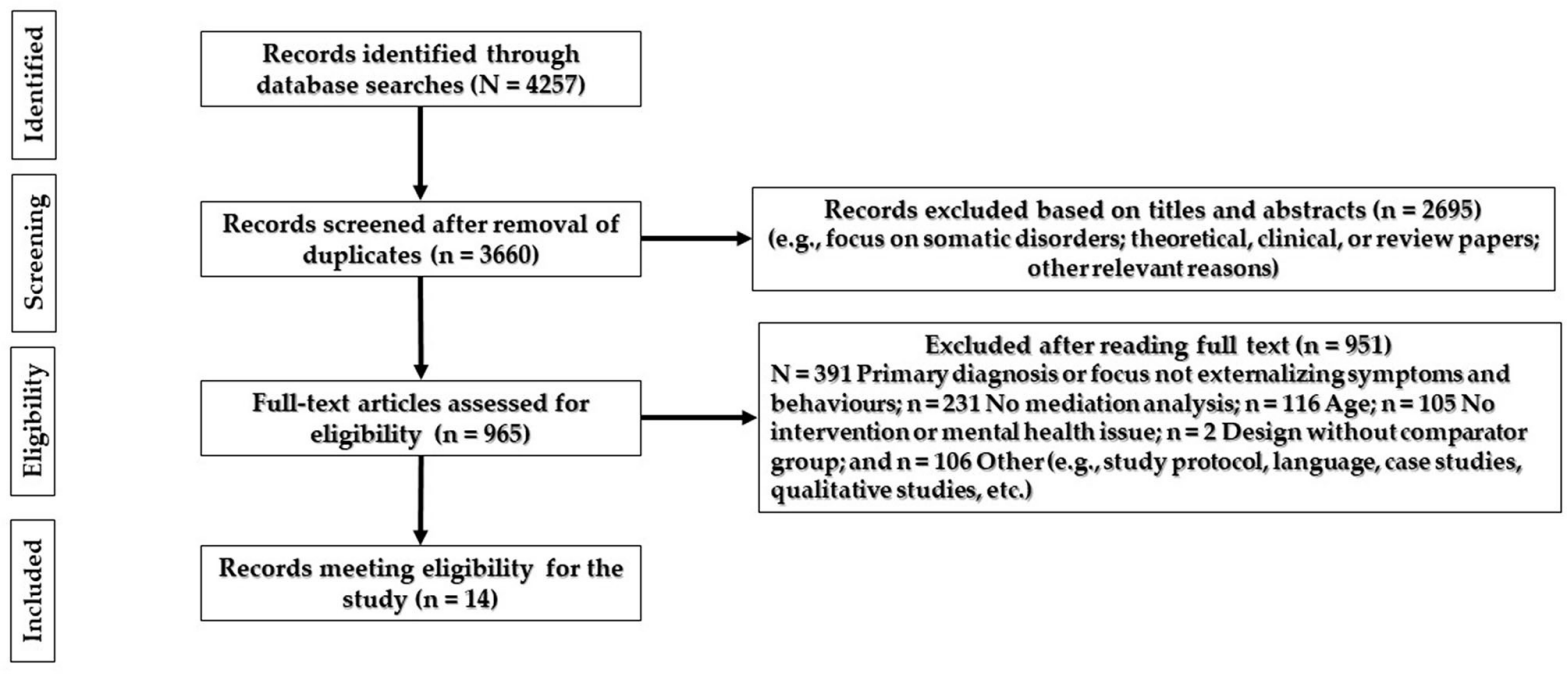
PRISMA flow diagram of the search.

Following the PRISMA guidelines (52), the flowchart presented in Figure 1 provides step-by-step details of our study selection process.

Literature search based on the search strings revealed that 4,257 articles were identified through database searches when the search string was applied. Two experienced researchers carried out an initial sample of articles identified through database search and developed a data file with authors, year, titles, and abstracts. Duplicate studies were eliminated.

Twenty experienced researchers divided into 10 pairs used an excel database of 3,660 articles built for this purpose. In the first stage, the screening was carried out using the titles and the abstracts of the papers. Each member of each pair screened titles and abstracts and excluded studies that clearly did not meet the inclusion criteria. Each pair established consensus about which studies to exclude. A total of 2,695 articles were excluded based on titles and abstracts.

In the second stage of the screening process, the remaining 965 studies were divided among 10 pairs of researchers. Each member of each pair reviewed the full text and discussed disagreements, and when consensus could not be reached, a third reviewer was involved in the decision. The reasons for exclusion were registered. Next, the full-text versions of potentially relevant studies were retrieved and examined in detail for eligibility by part of the COST Action TREATme team. Differences regarding study selection were resolved by discussion in team meetings. In the end, an independent researcher performed a quality cheque by assessing every fifth paper that was excluded in order to assess for reliability between individual rater pairs. A total of 951 were excluded, with 14 original articles in the final review.

### Data Extraction Processes

Two independent researchers extracted the data in pairs, and differences were discussed together. The categories for the extraction of data from the selected articles were as follows: specific symptoms, participants (age group, mean age, and standard deviation), setting of the intervention, design of the study, studied interventions, description of the comparator, times of assessment, follow-up(s) period(s), outcomes, results of each mediator in each outcome, and risk of bias.

### Strategy for Data Synthesis

We performed a narrative review of the results from the included studies. After reading the results of the studies, we identified the different types of mediators by the 10 pairs of researchers. They came together to compare and discuss the differences in the synthesis. Then, mediators were classified into four categories: family, relational, behavioural, and emotional. A meta-analytical pooling was not feasible because of overlapping mediator constructs and a small number of studies per type mediator.

### Risk of Bias and Quality Assessment

The 14 included studies were evaluated according to the quality of the evidence. Since no standard form for evaluating mediation studies has been established, methods of testing mediation effects were assessed according to the general criteria for identifying mediators of psychosocial interventions in research (53, 54). Namely, we considered (1) the sample size and study power, (2) validity and reliability of the measure used to assess the mediator, (3) temporality criteria and multiple assessments of the mediator, (4) comparison of theory-driven with non-specific mediators, and (5) whether there was manipulation of mediator dosages and their influence on the outcome. The Mixed Methods Appraisal Tool (55) was used for the risk of bias and the methodological quality assessment of randomised controlled trials.

## Results

### Study Characteristics

The PRISMA flow chart outlines the process of exclusion (see Figure 1) with the 14 studies, which met the eligibility criteria and were included in this systematic review.

A total of 12 of 14 experimental studies (56–67) examined samples with externalising behaviours as the primary diagnoses. In addition, there was one prevention study amongst 6th to 8th grade students (68) and one study that did not have a formal externalising diagnosis that was also related to a prevention study (69). Within these two studies (68, 69), externalising behaviours or symptoms were at least one of the primary outcomes.

Table 1 provides a detailed overview of extracted data and study characteristics. All articles came from Western-culture countries: 10 from the USA, 2 from the Netherlands, 1 from Canada, and the last 1 from Australia. American selected papers reported samples with high Hispanic adolescents (*k* > 60%); however, the rest of the studies did not report the ethnicity of the participants.

**Table 1:** Study characteristics.

**Reference [*n*], Country**	**Diagnosis, participants (% males), *M*_age_ (*SD*), groups (intervention vs. comparator); Dropout (*n*); Name of Intervention**	**Mediator(s) (M_**1**_ to M_**n**_), measures of mediator**	**Outcome(s) and Significant Mediator(s)** **↑increment and ↓decrement on mediators** **Δ↑increment and Δ↓decrement 'mediators' effects on outcomes** **(1/2): partial mediation**	**Quality of assessment (+: yes; -: no)** **RCT** **Control Group** ***n* ≥40 per group** **Multiple Mediator** **Temporality** **Mediator post/follow up/not applicable (P/F-up/n.a.)** **Experimental manipulation of mediator**						
		**Statistical analysis of mediation**	**Not Significant Mediator(s)**	**1**	**2**	**3**	**4**	**5**	**6**	**7**
Dadds et al. (56), Australia	Complex Conduct Problems *N* = 195 (25%) *M*_age_ = 10.52 (2.51) ERT group (*n* = 87). Dropout = 0 TAU group (*n* = 109). Dropout = 0 Empathic-Emotion Recognition Training	Changes in emotion recognition (M_1_) (FACES) Empathy (M_2_) (GEM) General linear mixed models	Child Adjustment (SDQ)	+	+	+	+	-	-	-
			M_1_: N. S. M_2_: N. S.							
De Bruin et al. (57), Netherlands	Externalising, internalising, and sleep problems *N* = 116 (75%) *M*_age_ = 15.6 (1.6) GT (F-2-F) (*n* = 38) Dropout (0,1,1) IT (online) (*n* = 39) Dropout (1, 15, 6) CG (*n* = 39) Dropout (3, 15, 3) Three waves CBT: Group Therapy (GT) and CBTI Online Therapy Cognitive-Behaviour Therapy Insomnia (CBTI)	Insomnia symptoms (M_1_) (HSDQ)	Psychopathology Problems (YSR) ↓ M_1_ led to Δ↓ of Affective and Anxiety Problems ↓_(1/2)_ M_1_ led to Δ↓ ADHDs' behaviour problems	+	+	+	-	-	F_up_	-
		Mediation multilevel regression analyses	M_1_ with oppositional defiant problems (N.S.)							
Dekovic et al. (58), Netherlands	Externalising Problems *N* = 256 (73.44%) *M*_age_ = 16.02 (1.31) MST = 147 Dropout (*n* = 17) TAU = 109 Dropout (*n* = 16) Multisystemic therapy (MST)	Parental sense of competence (M_1_) (subscale of PSI) Positive discipline (M_2_) (PDI) Inept discipline (M_3_) (subscales of PCSYR)	Single fit factors of CFA of several indicators from specified instruments for: Externalising Problems (ExtP) (Externalising symptoms, Violent Offending, Property Crimes, ODD, and CD) Relationship Quality (RQ, responsiveness, acceptance, conflict and antagonism, communication problems, external “observers” ratings). ↑ M_1_ and ↑M_2_ led to Δ↑ of RQ and Δ↓ of ExtP	+	+	+	+	+	P	-
		Latent growth modeling (70)	M_3_ with RQ (N.S.)							
Eddy and Chamberlain (59), USA	Chronic and severe offenders *N* = 79 (100%) *M*_age_ = 14.9 (1.3) MTFC (*n* = 37) Dropout (7) GC (*n* = 42) Dropout (*N* = 18) Multidimensional foster care treatment (MTFC)	Positive adult-youth relationships (M_1_) Parental Discipline (M_2_) Parental Supervision (M_3_) Deviant Peer Association (M_4_) Based on Interviews	Antisocial Behaviours (AB) (Interviews) ↓M_1_, ↑M_2_, ↑M_3_ and ↓M_4_ led to Δ↓ of AB	+	+	-	+	-	P	-
		Joint significant test (71)	-							
Fosco et al. (68), USA	6th−8th graders. No diagnoses *N* = 593 (51.4%). *M*_age_ = not reported FCU (*n* = 386). Dropout not reported School as usual (*n* = 207). Dropout not reported. Family Check-Up (FCU)	Self-regulation (M_1_), Effortful Control Subscale (EATQ)	AB [11 items based on (72)]. Deviant Peer Affiliation [5 items, (72)]. Cigarette, alcohol and marijuana use [3 items, (72)]. ↑M_1_ led to Δ↓ risks of AB	+	+	+	+	-	P	-
		SEM	-							
Gonzales et al. (69), USA	No formal diagnosis. Prevention of substance use, internalising and externalising problems. *N* = 516 (49.2%) *M*_age_ = 12.3 (0.54) Family Intervention (*n* = 338) GC (*n* = 178) Family Prevention (Bridges/Puentes) Dropout (*n*_s_ = 47, 7) 2nd and 3rd waves vs. Control Group Dropout (*n*_s_ = 21, 1) 2nd and 3rd waves Bridges/Puentes	Collected items of several instruments: Effective Parenting (M_1_) Family Cohesion (M_2_) Adolescent coping efficacy (M_3_) School Engagement (M_4_)	From different versions of YSR: Substance use Internalising Problems Externalising Problems Others: (GPA) School Disciplinary Actions ↑M_1_, ↑M_2_, ↑M_3_, and ↑M_4_ led to Δ↓ of all outcomes under moderation factors	+	+	+	+	-	P	-
		PRODCLIN program	-							
Henggeler et al. (60), USA	Several criminal activities *N* = 84 (100%) *M*_age_ = 15.2 (1.4) MST (*n* = 43). Dropout (*n* = 0) TAU (*n* = 41). Dropout (*n* = 1) Multi-systemic therapy (MST)	Family cohesion (M_1_) (FAM-III). Peer relations (M_2_) (MPRI) Adolescent Symptomatology (M_3_) (RBPC) Parental Symptomatology (SCL-90) Adolescent Social Competence (M_4_) (subscale of CBCL)	Criminal activities and incarcerations ↑M1 led to Δ↓ of youth aggression in peer relations	+	+	+	+	-	n/a	-
		Hierarchical multiple regressions	M_2_, M_3_, M_4_, and M_5_ (N.S.)							
Henggeler et al., (61), USA	Antisocial Behaviour (AB) and Sexual Offending (SO) *N* = 127 (97.6%) *M*_age_ = 14.6 (1.7) MST (*n* = 67). Dropout (*n* = 6) TAU (*n* = 60). Dropout (*n* = 6) Multisystemic therapy (MST)	Lax discipline, caregiver (M_1_) Bad Friends (M_2_) Parental Supervision (M_3_) Parental Communication (M_4_) Peer Delinquency (M_5_) (Measured using subscales of PYS)	Antisocial behaviour measured by YSR and SDR Substance use measured by PEI Sexual Deviance and Risk Taking (SDRT) measured by ASBI. ↑M_1_ and ↓M_2_ led to Δ↓ of AB and SDRT	+	+	+	+	+	P	-
		PRODCLIN program	M_3_, M_4_, M_5_ (N.S.)							
Hogue et al. (62), USA	Substance-abusing adolescents and externalising symptoms (79% of sample met criteria for ED) *N* = 100 (81.0%) *M*_age_ = 15.47 (1.31) Individual CBT (*n* = 56). Dropout (not reported) MDFT (*n* = 44). Dropout (not reported) Multidimensional Family Therapy (MDFT). Total dropout: at post, 26% were missing on each outcome. At follow-up, 32% were missing the drug use variable and 25% were missing the internalising and externalising variables	(M_1_) Adolescent Alliance (M_2_) Parental Alliance - in MDFT Group (M_3_) Interaction between adolescent and parent alliance, in MDFT Group (revised version of VTAS)	Drug use interview (TLFB), Externalising and internalising symptoms (CBCL and YSR); and treatment retention in CBT (Therapist logs). ↑M_1_ led to Δ↓ of Externalising symptoms only in MDFT group, at post and follow-up. ↑M_2_ led to Δ↓ of drug abuse and externalising symptoms in MDFT group, at post only. ↑ M_3_ led to Δ↓ of internalising symptoms at post in MDFT group.	+	+	+	+	+	P	-
		Hierarchical regressions	M_1_ in CBT group on outcomes (N.S.)							
Huey et al. (63), USA	Violent offenders with substance abuse *N* = 155 (83.5%) *M*_age_ = 14.6 (1.5) MST *n* = 82 Dropout (*n* = 25) CDA *n* = 73 Dropout (*n* = 13) Multisystemic Therapy (MST)	Adherence to MST (M_1_) (26-item MST) Family Functioning (MST group) (M_2_)/(FAM-III) Family Cohesion (CDA group) (M_3_) (FAM-III) Parent Monitoring (M_4_) (Monitoring Index of DPA) (M_5_) measured by RBPC	Delinquent Behaviour (DB) measured by SRD ↑M_1_, ↑M_2_; ↑M_3_, ↑M_4_ and ↓M_5_ led to Δ↓ of DB, only in MST group at post	+	+	+	+	-	P	-
		Latent variable path analysis and mediation analysis following (73).	-							
Jensen et al. (64), USA	Externalising symptoms, substance use, disruptive disorder, and internalising disorder diagnoses. *N* = 494 (50%) Adolescents and their mothers. Age not reported (approximately 15 years old) Dropout: not reported. No sample sizes reported by group. Two groups, family-focused intervention vs. control group. The times evaluation vs. Brief workshop	Mother–adolescent conflict (M_1_) (Adapted from a measure used in the PSFRP)	Externalising and internalising symptoms (YSR, ASR, CBCL) ↓M_1_ led to Δ↓ of Externalising Symptoms and substance use at T3 of Bridge Group	+	+	+	-	-	T_3_	-
		Path Analyses R Mediation program (74).	M_1_ with disruptive behaviours (N.S.)							
Pantin et al. (65), USA	Externalising Symptoms (drug, alcohol and unsafe sexual behaviours; externalising symptoms) *N* = 213 (63.85%). *M*_age_ =13.8 (0.76) Familias Unidas (FU) *n* = 109 CG *n* = 104. Community Control Families. Dropouts: nor reported by group Familias Unidas	Family Functioning: Parent Involvement (PPS) (M_1_) Positive Parenting (M_2_) (PPS DPS) Family Support (FRS) (M_3_) Parenting Adolescent Communication (M_4_) (PACS) Parental Monitoring (M_5_) (PRPGP)	SBI, DISC ↑M_2_, ↑M_4_, and ↑M_5_ led to Δ↓ on Externalising symptoms, substance use, and unsafe sexual behaviours in FU group	+	+	+	+	+	P	-
		Growth curve analyses	M_1_ and M_3_ (N.S.)							
Paquete and Vitaro (66), CANADA	Antisocial Behaviours *N* = 220 (88.73%) M_age_ = 10-days group 19.99 (2.41) 20-days group 19.54 (2.32) Sample 1 (10-day group) *n* = 101 Sample 2 (20-day group) *n* = 119. Dropout = 0 (both groups) Wilderness Therapy 10-day group. Vs. Wilderness Therapy 20-day group. Chance for Change Program	Interpersonal Skills (M_1_) (Ventura Trust) Accomplishment Motivation (M_2_) (Ventura Trust)	Externalising Symptoms Youth Antisociality (Ventura Trust) Length of days sessions had a positive indirect effect on lowering the level of “participants” antisociality, through the development of some interpersonal skills and accomplishment motivation.	+	+	+	+	+	P F_up_	-
Van Ryzin and Leve (67), USA	General Delinquency *N* = 166 (0%) Mean age = 15.31 (SD = 1.17) MTFC *n* = 81. No dropout reported GC = 85 No dropout reported Multidimensional Treatment Foster Care (MDFT)	Joint Significant Test (75) Delinquent peer affiliation (M_1_) (DFQ)	M_1_ and M_2_ (N.S.) Number of criminal referrals Number of days locked Self-report delinquency ↓M_1_ led to Δ↓ of all outcomes	+	+	+	-	-	P F_up_	-
		Path analyses	-							

*AB, Antisocial Behaviour; ACSBI, Adolescent Sexual Behaviour Inventory (76); ADHD, Attention deficit/Hyperactive Disorder; CBCL, Child Behaviour Check List (52); CBTI, Cognitive-Behavioural Therapy for Insomnia; CD, Conduct Disorder; CDA, Charleston Drug Abuse, SC, USA; CFA, Confirmatory Factor Analyses; DFQ, Describing Friends Questionnaire (77); EATQ, Early Adolescent Measure (78); ERT, Empathic-Emotion recognition Plus integrative family intervention; DPA, Delinquent Peer Affiliation (79); DISC, DISC Predictive Scales (80); FACES, Family and Child Experiences Survey (81); FAM-III, Family Assessment Measure (82); FCU, Family Check Up; GEM, Griffith Empathy Measure (83); GPA, Grade Point Average; HSDQ, Holland Sleep Disorder Questionnaire (84); IT, Internet Therapy; MDTF, Multidimensional Treatment Foster Care; MPRI, Missouri Peer Relations Inventory (85); N.S., Not significant; ODD, Oppositional Defiant Disorder; PACS, Parent–Adolescent Communication (86); PDI, Parenting Dimensions Inventory (87); PCSYR, Psychological Control Scale, Youth Report (88); PEI, Personal Experience Inventory (89); PPS, Parenting Practice Scales (90); PSI, Parenting Stress Index (91); PRPGS, Parent relationship with peer group scale (92); PSFRP, Penn State Family Relationships Project; RBPC, Revised Behaviour Problem Checklist (75); SBI, Sexual Behaviour instrument (93); SCL-90-R, Symptom Checklist (94); SDQ, Strengths and Difficulties Questionnaire (95); SEM, Structural Equation Model; SRD, Self-report Delinquency Scales (96); TAU, Treatment as Usual; TLFB, Alcohol Time Line Follow Up (97); VTAS, Vanderbilt Therapeutic Alliance Scale (98); YSR, Youth Self Report (99)*.

With regards to gender, the majority of the studies reported males mainly, with an average percentage of 74.05% male individuals (ranged from 51.4 to 100%); except one with 100% of female participants (67), another with 25% of males (56), and another one (64) with a balanced gender distribution (50%); however, these studies were preventive interventions on externalising symptoms.

The average age of all participants was 14.85 years (*SD* = 1.39, and ranged from 6 to 30 years old). However, two studies did not accurately report the mean age of participants by group or total; they only reported the school grades that indicated an adolescent sample.

Table 2 reports the name of intervention, format, mode of delivery, and setting of the selected studies.

**Table 2:** Characteristics of the interventions in the selected articles.

**References**	**Name**	**Type**	**Format**	**Delivery**	**Setting**
Dadds et al. (56)	ERT + IFI	SYS	IND/FAM	F-2-F	Outpatient
De Bruin et al. (57)	CBT_insomnia_	CBTI	IND	F-2-F/OL	Outpatient
Dekovic et al. (58)	MST	SYS	FAM	F-2-F	Outpatient
Eddys et al. (59)	MTFC	SYS + CBT	FAM/IND/GRO	F-2-F	Outpatient
Fosco et al. (68)	FCU	EDU + HUM	FAM	F-2-F	Outpatient
Gonzales et al. (69)	Bridges/Puentes	SYS	FAM/GRO	F-2-F	Outpatient
Henggeler et al. (60)	MST	SYS	FAM	F-2-F	Outpatient
Henggeler et al. (61)	MST	SYS	FAM	F-2-F	Outpatient
Hogue et al. (62)	CBT_individual_	CBT + SYS	IND	F-2-F	Outpatient
Huey et al. (63)	MST	SYS	FAM	F-2-F	Outpatient
Jensen et al. (64)	Bridges/Puentes	SYS	FAM/GRO	F-2-F	Outpatient
Pantin et al. (65)	Familias Unidas	SYS	FAM/GRO	F-2-F	Outpatient
Paquette et al. (66)	Wilderness Therapy	EDU	GRO	F-2-F	Outpatient
Van Ryzin and Leve (67)	MTFC	SYS + CBT	FAM/IND	F-2-F	Outpatient

*ERT, Emotion recognition training + Plus integrative family intervention; CBT, Cognitive-Behavioural Therapy; MST, Multi-systemic Therapy; MTFC, Multidimensional Treatment Foster Care*.

*Types: SYS, Systematic; EDU, Educational and preventive; HUM, Humanistic*.

*Format: IND, Individual; FAM, Familiar; GRO, Group*.

*Delivery: F-2-F, Face to Face; OL, On-line*.

Most psychological interventions were based on systemic approaches (*k* = 11), although three of them were blended with CBT (two with MTFC). Only one study was based on CBT only. Two studies used a psychoeducational approach, one in combination with a humanistic method. No other therapeutic modalities (e.g., psychodynamic or third wave therapies) were found to assess the change mechanisms and mediators matching our inclusion criteria.

With regards to the setting of the interventions, five of them included family members exclusively (58, 60, 61, 63, 68). Five others combined family and individual sessions (*k* = 5), two used individual sessions only (56, 66), two consisted of sessions in-group formats (64, 69), and one made use of all settings [group, individual, and family (59)]. Except for just one with an online group setting (56), the rest of the interventions were face-to-face. All studies were carried out with an outpatient population.

To assess ED, Achenbach and colleagues' YSR was used (57, 61, 66, 69) or CBCL (62) in most studies. However, different methods were used to determine externalising symptoms or behaviours, especially using items from other tools after implementing reliable confirmatory analyses (58, 68, 69). Most of the studies relied on ED outcomes using self-report measures. In contrast, some studies used interviews with parent and adolescents (59, 62) or derived ED directly from reports on delinquent activities and referrals (60, 67). There was a strong tendency to use confirmatory factor analyses to create a critical measure of ED through different items from different instruments, besides criminal or justice referrals (58, 61, 66, 68, 69).

The statistical analyses of mediation have changed in empirical research over time and thus varied in the reviewed studies. Most mediation analyses were based on different types of regressions analyses (57, 59, 61, 62, 66), two of them using the Baron and Kenny (71), and five studies included structural equations or path analyses to test mediational effects (61, 63, 64, 67, 68).

When it comes to Kazdin's criteria on quality of assessing mediation (see Table 1), only five studies met the temporality criteria, while none included the experimental manipulation of mediator. Although 11 out of 14 studies assessed multiple mediators, the vast majority of them compared mediators of the same kind (i.e., family or emotional mediators only).

### Risk of Bias and Quality Assessment for Process Research

Table 3 reports the risk of bias and quality assessments of the 14 included RCT studies. Although quasi-experimental studies were included as including criterion, no one was finally selected. All of them positively established research questions and collected the data according to the research questions. Nonetheless, regarding the performance of the randomisation sample, two studies had an unclear performance of the randomisation (62, 68), while the rest were appropriately randomised. All the comparator groups were assessed at baseline except one, which was unclear (68). More difficulties were found regarding the accomplishment of outcome assessors being blinded to the intervention provided: unclear (58, 64, 65, 67) and just one not accomplished (68). Finally, all studies satisfactorily completed the question regarding the adherence of participants, although two of them were unclear (62, 68).

**Table 3:** Risk of bias and quality assessment for process research.

**Questions**	**64**	**65**	**66**	**67**	**68**	**69**	**70**	**71**	**72**	**73**	**74**	**75**	**76**	**77**
Were there clear research questions?	+	+	+	+	+	+	+	+	+	+	+	+	+	+
Did the collected data allow to address the research questions?	+	+	+	+	+	+	+	+	+	+	+	+	+	+
Was randomisation appropriately performed?	+	+	+	+	±	+	+	+	±	+	+	+	+	+
Were the groups comparable at baseline?	+	+	+	+	±	+	+	+	+	+	+	+	+	+
Were outcome assessors blinded to the intervention provided?	+	+	±	+	-	+	+	+	+	+	±	±	+	±
Did the participants adhere to the assigned intervention?	+	+	+	+	±	+	+	+	±	+	+	+	+	+

*Yes (+), unclear (±), no (–)*.

*Dadds et al. (56); De Bruin et al. (57); Dekovic et al. (58); Eddy and Chamberlain (59); Fosco et al. (68); Gonzales et al. (69); Henggeler et al. (60); Henggeler et al. (61); Hogue et al. (62); Huey et al. (63); Jensen et al. (64); Pantin et al. (65); Paquete and Vitaro (66); Van Ryzin and Leve (67)*.

### Mediators

Overall, 37 potential mediators were examined, of which the majority (*m* = 23; 62.16%) were significant. Intervening factors or mediators with a partial indirect effect on outcomes are represented as “1/2” in Table 1. We found different types of mediators in the studies; however, most of them were related to family process or parenting styles. The letter “*m*” represents the number of times that a particular mediator has been identified in the different selected studies.

#### Family Mediators

These types of mediators represented the largest group (*m* = 22; 61.11%). Both family and parenting mediators were shown to be significant−16 out of 22: Family cohesion (*m* = 3), parental monitoring (*m* = 2), positive youth–adult relationships (*m* = 1), parental discipline (*m* = 1), parenting sense of competence (*m* = 1), lax caregiver discipline (*m* = 1), mother–adolescent conflict (*m* = 1), positive discipline (*m* = 1), positive parenting (*m* = 1), parent alliance (*m* = 1), effective parenting (*m* = 1), and parent–adolescent communication (*m* = 1). The following family-related mediators were reported to be non-significant (all *m* = 1): parent involvement, inept discipline, family support, parental communication, and parent symptomatology. Results from parental supervision were inconclusive with one study reporting significance whereas the other did not (*m* = 2).

#### Relational Mediators

With regards to relational-functioning mediators (*m* = 8, 21.62%), three of eight mediators were identified as significant: deviant peer association (*m* = 1), delinquent peer affiliation (*m* = 2), and bad friends (*m* = 1). Non-significant relational mediators were interpersonal skills (*m* = 1), peer relations (*m* = 1), peer delinquency (*m* = 1), adolescence social competence (*m* = 1), and adolescent alliance (*m* = 1).

#### Behavioural Mediators

Findings of behavioural mediators (*m* = 4, 10.81%) that were significant were as follows: school engagement, insomnia symptoms, and self-regulation behaviours. Accomplishment motivation (*m* = 1) was not significantly related to outcomes.

#### Emotional Mediators

Three potential mediators (6.46%) related to relational emotion processes have been tested: coping efficacy (*m* = 1) proved to be significant, while the change in emotion regulation (*m* = 1) and empathy (*m* = 1) did not.

No cognitive mediators were investigated in the selected papers of this systematic review. Importantly, all investigated mediators were non-treatment-specific.

## Discussion

To our knowledge, this is the first comprehensive systematic review that evaluated mediation studies in various forms of treatment of ED in adolescents. A total of 14 mediation studies with 3,314 participants were included, which investigated 37 different mediators in nine psychological interventions based on four theoretical foundations (systemic, CBT, educational, and humanistic).

A positive picture was gained regarding the risk of bias and study quality assessment. Although only eight studies met all the criteria for RCT, four additional faced only one problem—the absence of assessors' blindness. This result may suggest that the standards for performing RCTs are well-established and satisfyingly applied in the psychotherapy field. However, standards for mediation studies with respect to Kazdin's criteria (100) were low, which might be related to the fact that most studies were not designed as mechanism of change studies but efficacy studies in which mediation analyses were secondary research questions. Two main problems have been identified in the mediation designs: only one-third of studies included temporal precedence of the mediator, while none of studies performed experimental manipulation of mediator. The low quality of mediation testing questions the role of the proposed mediators in the respective mechanisms of change since causality could not be established and this means that changes in the putative mediators are mainly associated with the outcomes change. However, the temporal chain remains unclear (i.e., what changes first, outcome, or mediator).

With regards to outcome measures, the lack of a consensual criterion for assessing externalising behaviours was demonstrated. Namely, several studies (58, 60, 65, 66, 68) used various items from different self-report instruments to statistically develop an adjusted and reliable CFA to represent externalising behaviours. Others used objective, and hypothetically more robust measures of externalising behaviours such as interviews for testing antisocial behaviours (59, 62), criminal activities and incarcerations (60), and the number of criminal referrals and days locked up (67). The rest applied the externalising self-reports that are well-known and widely used in the literature. Thus, there was possible variance due to the evaluation method in outcomes (self-report vs. objective measures) that may have influenced the absence of significant results and would have required different statistical techniques, such as the multimethod–multitrait statistical approaches (53). Besides, some of the results might be compromised by the absence of clear clinical cutoff points in the instruments used for the assessment of externalising behaviours (50), while this was not the case for measuring depression with the Beck scale [see (13)].

The majority of psychological interventions were based on systemic principles (38). Hence, it appeared that systemic (family) psychological approaches were widely used in psychotherapy for treating externalising behaviours among adolescents (38) and are also focusing on establishing change mechanisms. Nonetheless, our systematic review points out that these interventions focused more on family-based treatment than on a theoretical systemic therapy orientation. Three randomised interventions were blended with CBT approaches (59, 62, 76), and eight used systemic theoretical mechanisms only (56, 58, 60, 61, 63–65, 69). They were presented in different formats: family (56, 58–61, 63–65, 67–69), group (59, 64–66, 69), and individual (59, 64–66, 69), and all systemic approaches were delivered face-to-face. Interestingly, no psychodynamic nor third-wave therapies were identified among these studies.

The review's main findings were related to the mediators that have been selected and proven to be significant. Family variables were found to be the largest group of investigated mediators (22 out of 37). The following groups are composed of a range of different relational (8 out of 37), behavioural (4 out of 37), and emotional (3 out of 37) mediators. No cognitive mediators were investigated. The lack of testing multiple mediators from different categories (e.g., family-related with emotional) was noted (see Table 1). Furthermore, none of the significant or non-significant mediators were treatment-specific and results in all these mediator categories showed both significant and non-significant results. We expect that some results are possibly coincidences (false-positive and false-negative) due to insufficient powering and not following Kazdin's recommendations.

With regards to the type of mediators, findings indicated that treatment approaches were mostly interested in evaluating mediators related to changes in the family system. Hence, *family mediators* were the largest group. Thus, they were closely related to the systemic therapeutic orientation and its findings (38). The commonalities observed with mediators showed how interventions improved family-based relationships (family cohesion, positive youth-adult relationships, lax caregiver discipline, mother-adolescent conflict, positive parenting, and parent-adolescent communication) and/or how-to-implement positive parenting (parental monitoring, parental discipline, parenting sense of competence, positive discipline, and effective parenting). These mediators were related to the presumed systemic mechanisms of change, which ranged from ineffective or negative parental styles to positive or more effective ones (58, 59, 62, 63, 65). Besides, studies also based on CBT approaches (59, 62, 67) included mechanisms of parental monitoring aimed at two primary purposes: to control/decrease risky peer relations (59–61, 63, 67) or/and to improve parental discipline (58, 59, 62, 64, 65). Both aim to help adolescents consider the consequences of their choices (especially with delinquent peers) before making decisions (1, 36, 57, 61, 88, 89, 95). These were followed by allowing adolescents to bear the consequences of poor decisions without bailing them out (5, 41, 60, 101, 102). Three studies (59, 61, 68) included family cohesion measures, i.e., strength of the emotional bonds between family members' mutual support under distressed environments and situations (103). Three studies demonstrated that increasing family cohesion might decrease ED (60, 63, 69). Therefore, it can be summarised that psychological ED treatments for adolescents often focused on these two mechanisms of change, i.e., increasing family relationships and improving effectiveness of parenting. Indeed, the majority of family-related mediators turned out to be significant in both domains of parenting and parent–adolescent relationship (16 out of 22). This points toward a putative change mechanism in the treatment of ED independent of the treatment approach.

Furthermore, our systematic review identified studies with *relational mediators* as the second largest group of mediators; however, only three out of eight proved to have significant effects. Significant relational mediators were related to the negative influence from peers adolescents with ED keep: deviant peer association, delinquent peer affiliation, and bad friends. Maintaining such relationships increases the likelihood of disruptive, delinquent or aggressive behaviour in adolescents with externalising symptoms (63, 67, 79). However, two studies investigating the influence of delinquent peers did not find a mediation effect on outcome. Furthermore, two studies reported on non-significant influences of social competences and also the therapeutic alliance.

Concerning *behavioural mediators*, two educational studies, which were oriented to decrease antisocial behaviours, were also reviewed (66, 68). One of them was a preventive study without a significant mediator (self-regulation) (68), and another had non-significant effects on lowering the level of participants' antisocial behaviour through the development of interpersonal skills and accomplishment motivation (66). Another rather unexpected significant mediator was reducing insomnia symptoms (CBT, individual and online settings), leading to lower internalising and externalising symptomatology (56).

Our review demonstrated a striking lack of studies on *emotional mediators* in research on treating externalising symptoms (100) as only three studies included mediators relating to emotional changes. Changes in empathy and emotion regulation turned out to be not significant in two studies, although increasing adolescents' emotional abilities is assumed to be a plausible mechanism that has been observed to improve the social (101, 104) and personality functioning (102, 103) of adolescents. The lack of emotional mediators as well as the absence of cognitive mediators may be a reflection of reductionist change theories in externalising disorders or the lack of effective treatments aside from family therapy that target at the system level more than on the adolescent him/herself.

There are some limitations to be considered when it comes to the methodology and findings of this systematic review. Firstly, we used general research terms and did not focus on externalising disorders in the systematic search—although it is expected that this kind of strategy would reveal any adolescent population in the psychotherapy process, more specific search terms would find at least, to some extent, different results. Second, the inclusion of only English literature could lead to some studies being excluded and some cultural biases. Thirdly, in order to obtain the most valid and reliable data, we excluded all the non-RCT studies that may limit or narrow the interpretability of available data. Hence, we found mainly US-based studies—and primarily systemic. Moreover, this review discussed the mediators that have been investigated so far, which turned out to be selective and biassed by the therapeutic school that was investigated. Finally, we aimed to give a narrative synthesis of the mediators. Due to their nature and variety, we were not able to conduct a meta-analysis and thus the theories of change specifically tested within the studies and related to specific therapies were not carried out.

Another issue to consider is the overlapping nature of mediators. In some ways, the types of mediators share characteristics. For example, family cohesion has both familial, relational, behavioural, and emotional connotations (104–106).

## Conclusions

Due to a lack of methodological quality in mediation designs, as stated above, results should be interpreted tentatively.

After reviewing the existing studies that followed an RCT design, therapy-related biases and restrictions in the mediator selection process were found. Restrictions may also be related to a narrow clinical change theory of ED. As a result, emotional and cognitive mediators were strikingly neglected. Furthermore, some inconsistences regarding the mediators' significance were identified. Namely, several family and relational mediators were significant in some studies and not in others, which prevented us from developing an explanatory pattern with more strength. Therefore, with these caveats in mind, our conclusions are tentative pending further research on psychotherapeutic and mediator efficacy. In principle, it appeared that both adequate behavioural control of adolescents' peer behaviour and a better positive balance in their relationships with their parents seemed to buffer the effects of externalising behaviours in adolescents with ED.

We tried to derive three groups of recommendations for future research based on the open questions that emerged after the synthesis of findings. Firstly, future studies should extend the existing knowledge by investigating other plausible mediators (i.e., including other emotional mediators, cognitive mediators). Also, the statistical comparisons of explanatory power of different mediators and investigation of their (complex) relationship is needed. Furthermore, all psychotherapy schools still face the challenge to explain how they work since no data on treatment-specific mediators has been found. Moreover, some therapeutic schools, like psychodynamic or third-wave therapies, so far have not been engaged in examining potential mediators of their ED treatment. CBT treatments have not been studied with regard to cognitive mediators either. Furthermore, within systemic and family therapy approaches, it would be interesting to explore further mechanisms (for instance adolescent's emotional or cognitive capacities) that are built during the therapy process and that would be effective in preventing ED after the adolescent develops more autonomy and starts to live an independent and separate life from their family. Moreover, it would be important to examine which mediators are significant in adolescents with severe ED (e.g., those in correctional institutions) and whose families are not available or have no contact or influence on the adolescents' behaviour.

The second group of suggestions relates to research practises and is based on studies' methodological limitations that were recognised in this review. Namely, several design issues could be improved in order to have stricter and more rigorous testing of mediation according to established criteria. Also, a greater consensus on how to assess externalising behaviours that represents the common symptoms of the different mental disorders that belong to the ED would enable more reliable and valid conclusions. Looking into gender as a moderator of mediation effects could be another challenging question addressed in the future.

Finally, additional efforts could be made in order to understand the relationship between internalising and externalising symptoms. Similarly, the use of personality functioning as a construct consisting of self and interpersonal domains instead of externalising vs. internalising could be used to avoid stigmatisation during adolescence.

## Data Availability Statement

The raw data supporting the conclusions of this article will be made available by the authors, without undue reservation.

## Author Contributions

JM, ST, MRH, CPM, AS, EH, SR, and SP drafted the first and second version of the manuscript. All co-authors provided a substantial contribution to the conception and design of the work by developing the research questions, the search string, and carrying out the stage 1 screening (SC-C, ST, EH, AS, SP, JV, AA, RB, DD, YI, JM, FM, CPM, MRH, JR, SS, TSP, RU, CS, and PM-P). The current manuscript was corrected and finally approved by the other authors (JV, AA, RB, DD, YI, SC-C, PM-P, FM, CPM, MRH, JR, SS, TSP, RU, and CS). RU coordinates the overall COST initiative. All authors agree to be accountable for all aspects of the work in ensuring that questions related to the accuracy or integrity of any part of the work are appropriately investigated and resolved.

## Funding

This publication is based upon work from COST Action: CA16102, European Network on Individualised Psychotherapy Treatment of Young People with Mental disorders, supported by COST (European Cooperation in Science and Technology). https://www.cost.eu/actions/CA16102/. The publication fee was paid by Heidelberg University (Germany) and Universidad de Càdiz (Spain).

## Conflict of Interest

The authors declare that the research was conducted in the absence of any commercial or financial relationships that could be construed as a potential conflict of interest.

## Publisher's Note

All claims expressed in this article are solely those of the authors and do not necessarily represent those of their affiliated organizations, or those of the publisher, the editors and the reviewers. Any product that may be evaluated in this article, or claim that may be made by its manufacturer, is not guaranteed or endorsed by the publisher.
